# Find and fuse: Unsolved mysteries in sperm–egg recognition

**DOI:** 10.1371/journal.pbio.3000953

**Published:** 2020-11-13

**Authors:** Enrica Bianchi, Gavin J. Wright

**Affiliations:** 1 Cell Surface Signalling Laboratory, Wellcome Sanger Institute, Cambridge, United Kingdom; 2 Department of Biology, Hull York Medical School, York Biomedical Research Institute, University of York, Wentworth Way, York, United Kingdom

## Abstract

Sexual reproduction is such a successful way of creating progeny with subtle genetic variations that the vast majority of eukaryotic species use it. In mammals, it involves the formation of highly specialised cells: the sperm in males and the egg in females, each carrying the genetic inheritance of an individual. The interaction of sperm and egg culminates with the fusion of their cell membranes, triggering the molecular events that result in the formation of a new genetically distinct organism. Although we have a good cellular description of fertilisation in mammals, many of the molecules involved remain unknown, and especially the identity and role of cell surface proteins that are responsible for sperm–egg recognition, binding, and fusion. Here, we will highlight and discuss these gaps in our knowledge and how the role of some recently discovered sperm cell surface and secreted proteins contribute to our understanding of this fundamental process.

## Abstract 2

Fertilisation is the challenging process whereby cells from two individuals fuse to generate a new, genetically distinct organism of the same species. This Unsolved Mystery article explores the molecular mechanisms underlying sperm–egg interaction and fusion, a fascinating topic that is under increasing investigation.

## Introduction

**Fig 1:**
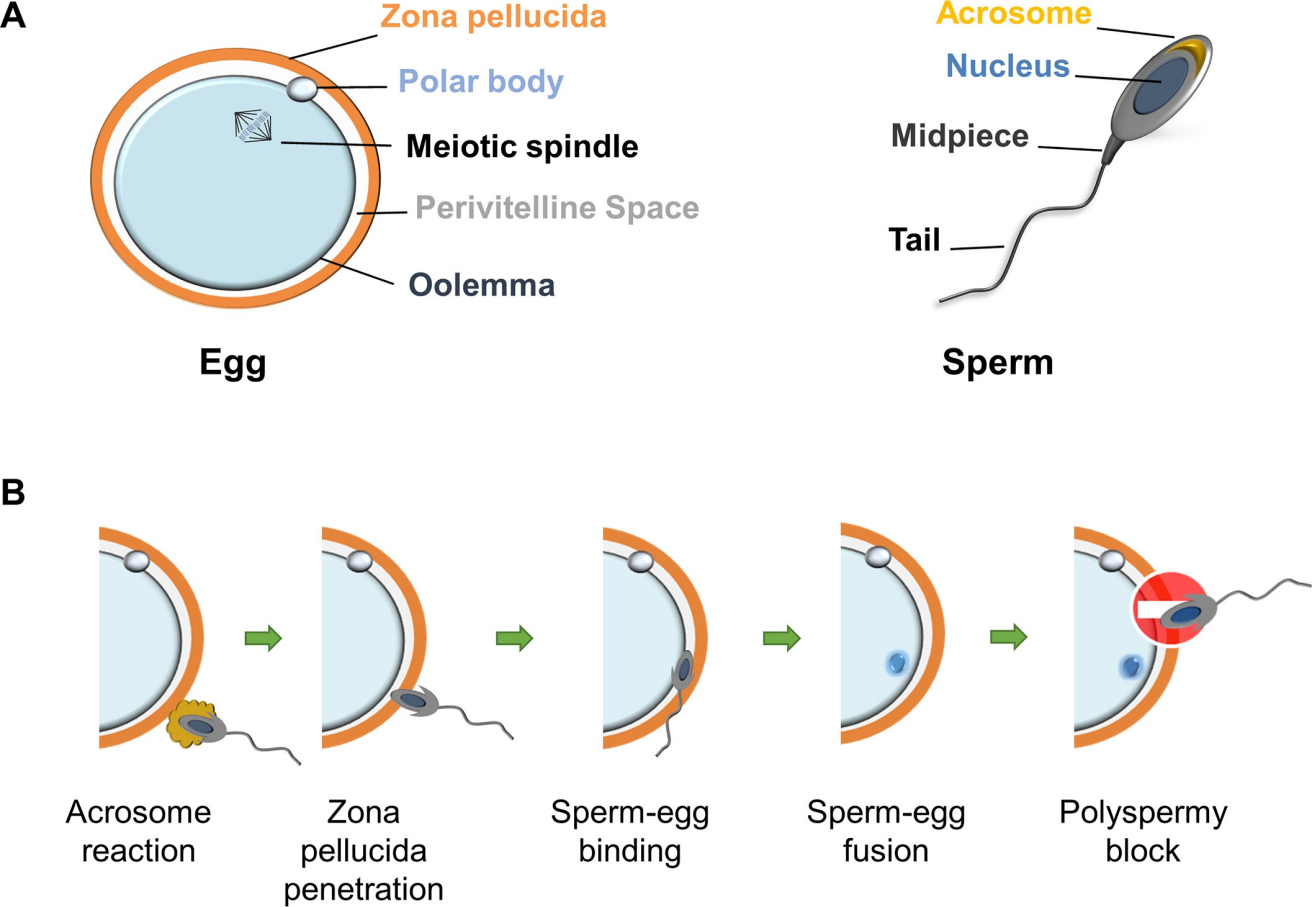
Schematic of mammalian gametes and the different stages of fertilisation. (A) Prior to fertilisation, mammalian eggs are held at metaphase II of the second meiotic division with the chromosomes aligned on the metaphase plate; half of the maternal DNA has been extruded and held within the polar body during meiosis I. The oolemma overlaying the meiotic spindle is devoid of microvilli, and sperm do not adhere or fuse in this region. Note that the egg and sperm are not drawn at scale; the sperm head is 5–10 times smaller than the egg. (B) Fertilisation is separated into a series of distinct stages. The acrosome reaction releases enzymes and exposes sperm ligands (such as IZUMO1) that were previously sequestered within the sperm head, and only acrosome-reacted sperm can pass through the ZP and fertilise the egg. Binding and fusion are regarded as separate events because there is evidence that they can be genetically and experimentally distinguished. The changes in the egg induced by fertilisation reduce the ability of sperm to fuse with an already fertilised egg in a mechanism known as the polyspermy block. The modifications occur at both the egg membrane and the ZP (for extensive reviews on this subject, see [4–6]). ZP, zona pellucida.

Fertilisation is the union of 2 haploid cells—the egg and sperm—to create a new diploid organism that ensures the propagation of genetic information from one generation to the next. The egg is one of the largest cells in mammals and is protected by a glycoprotein matrix, which appears as a semitransparent ring under the microscope and is consequently named the zona pellucida (ZP), from the latin ‘zona’, meaning belt or ring, and ‘pellucidum’, meaning transparent. The egg itself is surrounded by a membrane referred to as the oolemma and in mammals is characterised by a large number of short protrusions (microvilli), although these are absent in the region overlying the meiotic spindle, which is also marked by the presence of the polar body. Sperm have a distinctive morphology comprising 3 regions: the head, the midpiece, and the tail. The head contains the nucleus with the paternal DNA and an intracellular membrane-bound organelle, named the acrosome, situated above the nucleus. The midpiece contains mitochondria, which generate the chemical energy then transduced by the tail to propel the sperm in the female reproductive tract (Fig 1A).

Fertilisation has fascinated biologists for centuries, and the development of in vitro fertilisation (IVF) techniques in mammals have enabled a very detailed cellular description of how the sperm and egg behave and interact. Based on these microscopic observations, fertilisation can be conceptually separated into 4 consecutive events (Fig 1B). Firstly, the acrosomal membrane fuses with the sperm plasma membrane, a process known as the acrosome reaction. This event releases enzymes and cell surface ligands that were previously sequestered within the sperm head [1,2]. Secondly, the sperm must penetrate the ZP to gain access to the perivitelline space: a conspicuous gap between the egg and the ZP. Thirdly, the acrosome-reacted sperm must recognise and adhere to the oolemma, which is then followed by the fusion of the sperm and egg membranes so that two cells become one; a new diploid organism has been created, and fertilisation has been achieved [3]. Finally, the fertilised egg must prevent additional sperm fusions to avoid creating a nonviable polyploid embryo, and this is achieved by altering both the ZP and oolemma so that they are less receptive to additional incoming sperm [4–6]. In this Unsolved Mystery, we will focus on the cellular interactions of the sperm and egg and especially the molecules involved in sperm binding to the ZP, sperm binding to the oolemma, and cell membrane fusion; for each step, we will highlight where the molecular mechanisms involved remain an unsolved mystery.

Despite the detailed cellular description of this process, our understanding of the molecular mechanisms involved has been impeded by some unique experimental challenges [7]. Sperm and eggs are terminally differentiated cells that survive for just a short time outside of the body and rapidly degenerate and die if fertilisation does not occur. Mature sperm are produced in vast quantities throughout the male’s adult life and during the last stages of spermiogenesis become transcriptionally and translationally silent [8,9]. By contrast, a limited number of oocytes are present in the ovary, and very few of them eventually mature and are released or ovulated during the life span of the female. In mammals, oocytes are a very rare cell type, limiting the amount of biological material available, and there are understandably very strict ethical restrictions that prevent mixing human sperm and eggs for experimental analysis. Furthermore, studying the role and molecular interactions of membrane-embedded cell surface receptors is difficult due to their amphipathic character and the typically highly transient nature of extracellular protein–protein interactions [10].

The development of IVF techniques in mammals was one of the major biomedical breakthroughs of the 20th century, opening the path to assisted fertility treatments [11,12] and providing scientists with an invaluable tool to investigate the cellular basis of sperm–egg interactions. For the first time, gametes could be observed and studied outside of the organism, and the molecular basis of this cellular recognition process could be easily investigated by adding exogenous molecules such as antibodies, cell extracts, and inorganic compounds [13–15]. Unfortunately, many candidates that were identified with these approaches turned out to be dispensable for fertilisation when their role was investigated using targeted gene disruption in mice [16]; for example, the role of *ADAM* (A Disintegrin And Metalloprotease)*1b/ADAM2*—a sperm surface heterodimer initially named fertilin [17]—was questioned when male gene-deficient mice were subsequently found to be fully fertile [18]. IZUMO1, named after a Japanese marriage shrine, is the only example of a sperm cell surface protein that was initially demonstrated to be essential for fertilisation by adding a monoclonal antibody recognising this protein in IVF assays [19] and whose role was unequivocally confirmed in gene-deficient mice [20]. The introduction of gene editing technologies in the 1980s represented an invaluable tool to investigate gene functions in model organisms [21], and the remarkable advances made over the last decade [22] have made the creation of gene-deficient mice much easier and have been systematically applied to investigate the role of a large number of potential sperm candidates, many of which were found to have no role in fertility [23,24]. Remarkably, in just a few months, 4 new genes that encode sperm cell surface or secreted proteins have been reported that are essential for male fertility: SPerm ACrosome membrane-Associated protein 6 (*Spaca6*), Fertilisation Influencing Membrane Protein (*Fimp*), Sperm–Oocyte Fusion required 1 (*Sof1*), and TransMEMbrane protein 95 (*Tmem95*) [25–28]. In this article, we will discuss 2 fundamental and yet enduring mysteries concerning sperm–egg recognition and place these recent discoveries into our understanding of fertilisation.

## How does the sperm recognise the ZP?

The ZP forms a protective coat around the egg and presents a major barrier for the sperm to access the egg cell membrane. In humans, this coat of extracellular matrix is made of 4 proteins (ZP1, ZP2, ZP3, and ZP4), and in mouse of 3 because *Zp4* is a pseudogene. In other mammals, different combinations of the same glycoproteins exist; for example, the ZP in pigs and cattle is composed of orthologues of ZP2, ZP3, and ZP4. The observation that sperm readily bind to the ZP of unfertilised eggs and yet are unable to bind the ZP of 2-cell embryos has prompted researchers to investigate which of the ZP proteins mediates this binding and acts as a sperm receptor. It is now clear that the N-terminal proteolytic cleavage of ZP2 reduces the ability of sperm to bind the egg [29–31], and domain swapping experiments in transgenic animals support the hypothesis that ZP2 is the sperm receptor [32]. After fertilisation, the egg is said to become activated, triggering the exocytosis of cortical granules to release an astacin-like metalloendopeptidase named ovastacin. The mutation of the ZP2 processing site or genetic ablation of the gene encoding ovastacin prevents ZP2 cleavage, resulting in the retention of sperm binding to the ZP even after fertilisation [33,34]. Because we do not yet have a candidate sperm ligand for ZP2, it remains a mystery whether the cleaved fragment of ZP2 is the actual sperm receptor or whether the structural changes resulting from the cleavage obscure or remove the sperm-binding site. Evidence suggesting that ZP3 and/or glycans are the ZP sperm receptor has also been obtained [35]; however, targeted gene deletion experiments in animal models did not settle the question because ZP2 and ZP3 are necessary for the formation of the ZP [29,36]. Notably, none of the new essential sperm proteins seem to be involved in ZP binding, as removal of the ZP does not overcome the infertility defect of gene-deleted sperm [25–28]. The crystal structure of ZP proteins might finally provide the long-awaited answer, and a recent structure of the ZP domain, a protein polymerisation module of approximately 260 amino acids found in many secreted proteins including all ZPs, suggests that the sperm-binding region might lie at the interface between the ZP2 and ZP3 subunits [37]. How sperm pass through the fibrillar network once they are bound to it remains an open question in the series of events leading to fertilisation. Novel insights were obtained from the structure of the sperm protein lysin and its egg-binding partner VERL (Vitelline Envelope Receptor for Lysin) from the marine mollusc abalone. Although there are no known homologs of lysin in vertebrates, the functional regions of ZP2 and VERL are structurally homologous, suggesting that similar mechanisms for binding and passage through the ZP might be adopted by species as different as molluscs and mammals [38,39].

## Do the recently identified sperm proteins mediate fusion or binding?

The gold standard method to assess whether a gene is essential for fertilisation is to test the fertility of a genetically modified animal lacking the gene of interest [40]. Based on this criterion, until recently only 3 genes were known to be required for fertilisation in mammals, each encoding a cell surface protein: CD9 (Cluster of Differentiation 9) and JUNO (named after the Roman goddess of marriage and fertility) in the egg [41–43] and IZUMO1 in the sperm [20] (Fig 1). Animals lacking these proteins have a characteristic phenotype; although they are able to produce sperm and eggs that are overtly normal in number, appearance, and behaviour, fertilisation fails at the final adhesion and fusion step.

**Fig 2:**
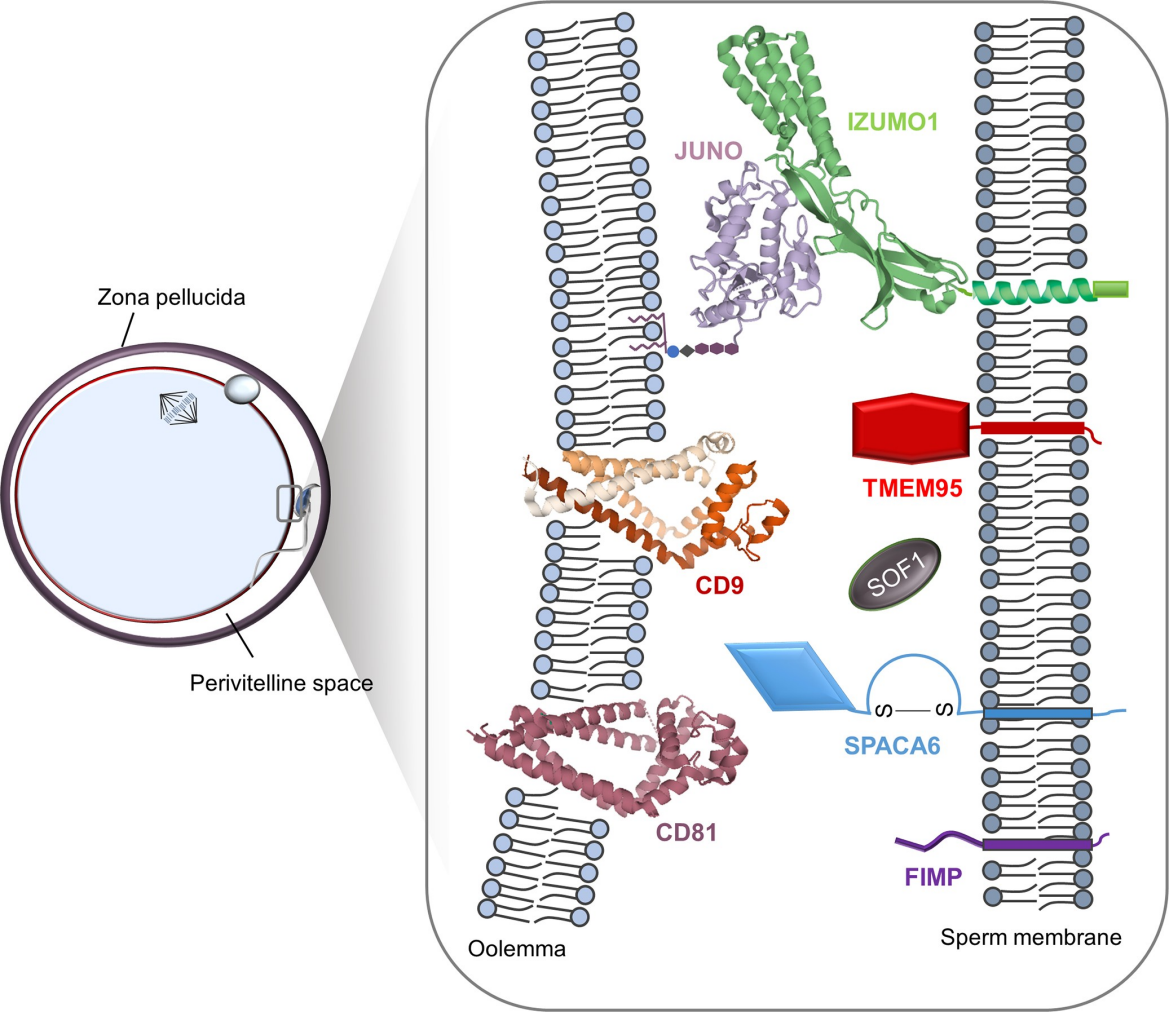
Cell surface proteins required for fertilisation in mammals. The binding of the GPI-anchored egg protein JUNO to the ectodomain of the sperm protein IZUMO1 ensures the adhesion of gamete cell membranes and is essential for fertilisation. Three single-pass membrane proteins expressed by sperm—SPACA6, TMEM95, and FIMP—are necessary for fertilisation. Like IZUMO1, the extracellular region of SPACA6 contains an immunoglobulin-like domain, whereas no structural data are available for TMEM95 and FIMP. The fourth sperm protein essential for fertilisation, SOF1, is a putative secreted molecule with an unknown structure. Two tetraspanins, CD9 and CD81, are also required for fertilisation, and there is no evidence that they directly interact with any of the other proteins. CD, Cluster of Differentiation; FIMP, Fertilisation Influencing Membrane Protein; GPI, Glycosylphosphatidylinositol; SOF1, Sperm–Oocyte Fusion required 1; SPACA6, SPerm ACrosome membrane-Associated protein 6; TMEM95, TransMEMbrane protein 95.

A thorough investigation of CD9-deficient eggs showed that the shape and distribution of their microvilli was altered [44], suggesting that this protein is involved in organising the overall architecture of the cell membrane and the other proteins embedded within it. CD9 belongs to a family of membrane proteins known as the tetraspanins that, as their name suggests, contain 4 transmembrane-spanning regions, which was recently confirmed by structural studies [45]. CD9 forms homophilic and heterophilic interactions as well as macromolecular complexes with other tetraspanins including CD81 [46], which is also expressed on eggs and the absence of which also reduces female fertility; remarkably, eggs lacking both CD9 and CD81 are sterile without affecting sperm–ZP penetration [47]. Tetraspanins have well-established roles in cell adhesion and signalling [48], which prompted a search for CD9 and CD81 interaction partners, but those identified subsequently turned out to be dispensable for fertilisation [49]. Some elegant biophysical measurements have shown that CD9 generates fusion-competent sites on the egg surface [50] with an accumulation of CD9 molecules at the sperm–egg interaction site that precedes the fusion event [51]. These observations are consistent with the ability of tetraspanins to organise the distribution and functional clustering on the plasma membrane by forming highly organised microdomains [52]. The other essential egg protein, JUNO, was identified as an IZUMO1 receptor by expression cloning of a mouse oocyte cDNA library [42]. IZUMO1 is a sperm cell surface ligand that was identified 9 years previously and shown to be essential for male fertility [20]. The 2 proteins form a receptor–ligand pair that is essential for sperm–egg adhesion (Fig 2), and the postfertilisation shedding of JUNO from the egg surface provides a plausible mechanism for the membrane block to polyspermy [42,53]. JUNO and IZUMO1 expression and binding has been confirmed in humans [54] and in other animals [55,56], suggesting their role is conserved amongst mammals. Heterologous expression of IZUMO1 and JUNO in neighbouring cells does not induce membrane fusion, spurring a search for other proteins known as ‘fusogens’ that are necessary for the final step in fertilisation. The existence of an additional egg receptor for IZUMO1 has been proposed in a model whereby the initial engagement of JUNO by IZUMO1 triggers IZUMO1 dimerisation, causing a protein disulfide isomerase-mediated conformational change that disengages IZUMO1 from JUNO, thus making it available to bind a second receptor and thereby bring the gamete cell membranes in close proximity [57]. This model needs further experimental evidence, as no other egg receptor other than JUNO has been identified for IZUMO1 and IZUMO1 lacks any structural homology with known fusogens [58] from other organisms, supporting the idea that this is an adhesion protein rather than a fusogen.

The use of CRISPR technologies has led to the recent and remarkable identification of 4 new sperm proteins that are essential for mammalian fertilisation (Fig 2). They are 3 membrane-anchored proteins, FIMP [28], SPACA6 [25,27], and TMEM95 [25,26], and a predicted secreted protein, SOF1 [25]. Similarly to IZUMO1, SPACA6 is a type I transmembrane protein with a short cytoplasmic C-terminus and an immunoglobulin (Ig)-like domain in the midst of its extracellular region. TMEM95, FIMP, and SOF1 are small proteins of less than 200 amino acids that are expressed highly in the testis. Male mice lacking any one of these 4 proteins phenocopied *Izumo1*-deficient males; they produce sperm of normal morphology and motility, and their passage of the ZP and binding to eggs were comparable with that of wild-type sperm. However, they failed at the very last step and did not fuse to the egg. One major difference between IZUMO1 and the 4 new proteins, however, is that heterologous cells overexpressing IZUMO1 are able to efficiently adhere to eggs, a property that is not shared by any of the new candidates, suggesting they have a minor or no role in sperm–egg recognition. Could any of these new candidates therefore fulfil the role of a fertilisation fusogen?

## Gamete fusogens: An evolutionary perspective

Because merging membranes is an energetically unfavourable event, it requires the action of a specific protein or fusogen. The assessment of a molecule’s fusogenic ability is often based on indirect measurements, usually the intracellular transfer of a marker between the 2 fusing cells. There is also a tacit assumption that the candidate fusogen maintains its activity when isolated from its native biological environment and expressed in a heterologous system. Like IZUMO1, SPACA6, FIMP, TMEM95, and SOF1 are not able to induce cell fusion either alone or together when assessed in a cell fusion assay [25–28]. These novel sperm proteins, therefore, seem unlikely to be the long-sought–after molecule that induces fusion in mammalian gametes. FIMP in particular does not seem to fit this role because male fertility was rescued by transgenic overexpression even though it was undetectable in 40% of acrosome-reacted sperm, suggesting that FIMP is not essential after the acrosome reaction and therefore not involved in sperm–egg fusion.

If they are not fusogens and are not involved in sperm–egg binding, could they have a cell-autonomous function in sperm, perhaps by forming complexes with other sperm proteins? During the acrosome reaction, a massive reorganisation of the sperm membranes relocates sequestered molecules like IZUMO1 to a specific region on the sperm surface that triggers fusion with the oolemma. It is conceivable that even a small imperfection in this relocalisation could produce a strong effect; however, light microscopy has shown that IZUMO1 relocalisation is not grossly affected when SPACA6, FIMP, TMEM95, or SOF1 are absent [25–28]. Nonetheless, more powerful microscopy techniques that can provide a more detailed spatiotemporal analysis of IZUMO1 localisation could provide data that are more informative. Could these novel proteins ensure IZUMO1 functionality? Cell binding assays and coimmunoprecipitation experiments suggest that they do not directly interact with IZUMO1, but the functional cooperation of SPACA6, TMEM95, SOF1, and FIMP has not yet been investigated thoroughly.

A different but related protein, SPACA4, represents a very interesting case from an evolutionary perspective, and a possible gamete fusogen SPACA4/sperm acrosomal membrane protein 14 (SAMP14) is a Glycosylphosphatidylinositol (GPI)-anchored protein displayed on the surface of acrosome-reacted sperm whose role in mammals has to be fully elucidated [59]. An antibody raised against the recombinant SPACA4 blocked the fertilisation of ZP-free hamster eggs by human sperm [60], but as we discussed above, the generation of genetically modified mice will be necessary to determine whether this is an essential protein. Notably, the orthologue of SPACA4 in zebrafish—a protein named Bouncer—is essential for sperm–egg recognition in this model vertebrate [59]. In zebrafish, Bouncer is displayed on the surface of eggs and ensures species-specific fertilisation, a biology that has more relevance for broadcast-spawning species than in internally fertilising mammals. The identity of the sperm receptor for Bouncer remains unknown, and if one exists, it will be important to understand whether its expression is also restricted to different gametes.

## Conclusion

Identifying the molecules required for fertilisation and understanding how they cooperate and interact to ensure sperm–egg recognition, binding, and fusion could lead to a substantial improvement of Assisted Reproductive Technologies (ARTs) not just in humans but also in endangered species whose survival may depend on such approaches. ARTs are also widely used in livestock production, in which more efficient protocols are a valuable asset. It could additionally lead to the development of better diagnostic tests for infertility and to new nonhormonal contraceptives.

### Concluding remarks on approaches and technical advancements

The identification of these new essential sperm proteins will undoubtedly be an important contribution towards a comprehensive molecular understanding of mammalian fertilisation, although further research is now needed to understand their precise mechanistic roles. Whether or not a fertilisation fusogen exists remains an important but unanswered question. In plants and algae, the use of genetic screens for fertilisation and fusion mutants has led to the discovery of HAP2 (Hapless 2) and is an example of how this issue may be tackled. HAP2/GCS1 (Generative Cell Specific 1) is a membrane protein essential for gamete fusion that is conserved in all lower eukaryotes except fungi [61–63] and has no homologs in vertebrates. HAP2 is structurally similar to EFF-1 (Epithelial Fusion Failure-1), a somatic cell fusion protein in *Caenorhabditis elegans*, and to viral class II fusion proteins [64], indicating that they might have evolved from a common ancestor [65]. We can conceivably suppose that systematic large-scale screens, together with the continuous improvement of cryo-electron microscopy techniques and the development of more efficient protocols to obtain gametes from somatic cells, will pave the way to new exciting discoveries.

## Abbreviations

*ADAM*: A Disintegrin And Metalloprotease
CD: Cluster of Differentiation
ART: Assisted Reproductive Technology
EFF-1: Epithelial Fusion Failure 1
FIMP: Fertilisation Influencing Membrane Protein
GCS1: Generative Cell Specific 1
GPI: Glycosylphosphatidylinositol
HAP2: Hapless 2
Ig: immunoglobulin
IVF: in vitro fertilisation
SAMP14: sperm acrosomal membrane protein 14
SOF1: Sperm–Oocyte Fusion required 1
SPACA6: SPerm ACrosome membrane-Associated protein 6
TMEM95: TransMEMbrane protein 95
VERL: Vitelline Envelope Receptor for Lysin
ZP: zona pellucida
